# DaVIE: Database for the Visualization and Integration of Epigenetic data

**DOI:** 10.3389/fgene.2014.00325

**Published:** 2014-09-18

**Authors:** Anthony P. Fejes, Meaghan J. Jones, Michael S. Kobor

**Affiliations:** Centre for Molecular Medicine and Therapeutics, Child and Family Research Institute, University of British ColumbiaVancouver, BC, Canada; Department of Medical Genetics, University of British ColumbiaVancouver, BC, Canada

**Keywords:** epigenetics, visualization, 450k methylation array, database, bioinformatics

## Abstract

One of the challenges in the analysis of large data sets, particularly in a population-based setting, is the ability to perform comparisons across projects. This has to be done in such a way that the integrity of each individual project is maintained, while ensuring that the data are comparable across projects. These issues are beginning to be observed in human DNA methylation studies, as the Illumina 450k platform and next generation sequencing-based assays grow in popularity and decrease in price. This increase in productivity is enabling new insights into epigenetics, but also requires the development of pipelines and software capable of handling the large volumes of data. The specific problems inherent in creating a platform for the storage, comparison, integration, and visualization of DNA methylation data include data storage, algorithm efficiency and ability to interpret the results to derive biological meaning from them. Databases provide a ready-made solution to these issues, but as yet no tools exist that that leverage these advantages while providing an intuitive user interface for interpreting results in a genomic context. We have addressed this void by integrating a database to store DNA methylation data with a web interface to query and visualize the database and a set of libraries for more complex analysis. The resulting platform is called DaVIE: Database for the Visualization and Integration of Epigenetics data. DaVIE can use data culled from a variety of sources, and the web interface includes the ability to group samples by sub-type, compare multiple projects and visualize genomic features in relation to sites of interest. We have used DaVIE to identify patterns of DNA methylation in specific projects and across different projects, identify outlier samples, and cross-check differentially methylated CpG sites identified in specific projects across large numbers of samples. A demonstration server has been setup using GEO data at http://echelon.cmmt.ubc.ca/dbaccess/, with login “guest” and password “guest.” Groups may download and install their own version of the server following the instructions on the project's wiki.

## 1. Introduction

With recent advances in technology, there has been a dramatic surge in the collection and publication of epigenomic data. In particular, population- and disease-based studies have focused on DNA methylation, the 5-methylation of cytosine bases. One of the many epigenetic mechanisms that cells use to regulate gene expression, it is the most accessible and easily studied. Typically, DNA methylation occurs at locations where cytosine residues are followed by guanine residues, called CpGs. The methyl group is placed on the 5 carbon of the cytosine by DNA methyltransferases and can be removed either actively or passively (Law and Jacobsen, 2010). DNA methylation is also highly tissue-dependent, such that tissue of origin is the factor that most affects DNA methylation pattern (Ziller et al., 2013). This means that it is vital to be able to compare findings in a specific study to data from both the same and different tissues, in order to properly interpret it in context. In addition to its role in gene expression, DNA methylation is known to take part in important cellular functions such as X chromosome inactivation and genomic imprinting (Robertson, 2005), and evidence is growing that it plays a major role in encoding the influence of the environment in the nature vs. nurture debate (Tobi et al., 2012; Mehta et al., 2013; Bind et al., 2014).

Given the increase in such evidence, it is perhaps not surprising that more and more Epigenome-Wide Association Studies (EWAS) studies are being conducted. Many of these new studies are taking advantage of the Infinium HumanMethylation450 BeadChip (Illumina, San Diego, CA), also known as the 450k methylation array. This platform is based on an array of probes that quantify the methylation status of over 450,000 positions in the genome (Bibikova et al., 2011).

EWAS studies are using this array to examine DNA methylation in different tissues, diseases, and populations, which leads to immense potential for cross-project meta-analyses. However, this type of analysis remains a challenge for researchers because of the difficulty of combining data from a variety of sources, which has translated into a scarcity of tools that provide researchers with the ability to interact with the data in an intuitive manner.

One of the main themes of recent bioinformatics web browsers has been the use of tracked data sets, where each data set is displayed individually. This allows a large number of individual results to be compared sequentially. The tracked model has been popularized in large part due to the track functionality of the UCSC Genome Browser (Kent et al., 2002). The ability to upload custom tracks and include the display of frequently accessed public data sets as tracks has deservedly made this platform one of the most commonly used in human genomic research, as well as for other supported organisms. This tracked method is used in several current DNA methylation databases, including the UCSC's own 450k track. For example, the Washington University epigenetics browser displays a novel visualization in the form of their methylC Track (Zhou et al., 2014), which yields a visual interpretation of a single DNA methylation data set for high-resolution DNA methylation. (e.g., Single base pair resolution from next generation sequencing methods.) In contrast, the Pancreatic Cancer Methylation Database (Nagpal et al., 2014) has opted to avoid use of the browser entirely, providing summary and statistical information for DNA methylation across cell lines and genes.

Despite the wide variety of visualization methods that are currently in use, there remain opportunities to develop novel tools. We have developed such a package, called DaVIE, which is capable of storing, retrieving, and visualizing DNA methylation data across projects. We believe that this type of database and browser based system can provide an excellent platform for making epigenetics data accessible to researchers with varying levels of experience with computational tools that are required to analyze and interpret DNA methylation data.

Once populated with in-house generated or public data, users can easily look up the DNA methylation status of specific genes of interest in a variety of tissues. In addition to creating opportunities to cross-check findings and identify outlying samples or tissues, DaVIE has the ability to promote hypothesis generation through its intuitive and informative interface.

## 2. Materials and methods

This application uses the following software packages:, MongoDB 2.4.10, python 2.7, pymongo 2.6.3 Django 1.6.1, django-mongo-auth 0.1.2, django-mongodb-engine 0.5.1, mongoengine 0.8.7, pyjade 2.0.3, pylint 1.1.0, pymongo 2.6.3, simplejson 3.3.0, svgwrite 1.1.4, and is deployed on Ubuntu server 13.10. Code developed for this project, as well as issue trackers and full documentation for deploying the software are all available on github at https://github.com/apfejes/epigenetics-software.

### 2.1. MongoDB database

DaVIEs web interface was built upon a custom designed MongoDB database, described in a separate manuscript currently under preparation. MongoDB databases use the JSON (ECMA, 2013) language for storing information. Thus, python's dictionaries and lists (or any nested combination of them) may be stored in the database and efficiently retrieved. This allows for data structures to be stored in the database that would otherwise not be easily created, maintained or retrieved using an SQL database.

The use of the JSON standard is an important requisite for the deployment of sample grouping and separation of data by probe in order to provide rapid responses to queries passed through the web interface.

### 2.2. Python

Code written in Python 2.7 is used to process and interpret the results stored in DaVIE, bridged by the PyMongo library (http://api.mongodb.org/python/current/), which allows a simplified interface for data retrieval and manipulation. The code is divided into modules, which include the django web interface, wrappers for the database and code used to generate the images displayed to the user.

### 2.3. Django webserver

The django web server is used to accept and reply to requests made to the software package, as well as to handle authentication and security through the MongoEngine (http://mongoengine.org/) package. Currently, users can be created through an Admin interface, which enables the creation of accounts that allow users to access pages within the web application that provide access to visualize, edit, and upload data. The PyJade module (https://github.com/SyrusAkbary/pyjade) is used to render Jade templates into HTML to generate the interface.

### 2.4. Scalable vector graphics

To visualize the data, the python code uses the svgwrite (https://bitbucket.org/mozman/svgwrite) package to generate Scalable Vector Graphics (SVG) images. This XML-based format is supported as a W3C standard, and is broadly implemented in many illustration programs as well as web browsers.

## 3. Results

The recent increase in EWAS studies has resulted in the production of large amounts of DNA methylation data, with a growing emphasis on the 450k array. Between in-house produced and GEO deposited data, there is immense potential for large-scale studies, but there remain challenges in combining data from different projects. We created the DaVIE database and visualization platform to allow quick and easy comparison of DNA methylation data across projects, tissues, or any other variable of interest that has been provided in the sample metadata. We have used DaVIE to identify outlier samples, observe trends in DNA methylation over projects and tissues, as well as easily examine the DNA methylation status of candidate genes from specific projects in the context of a large number of samples.

### 3.1. Data deposition

Data can be deposited into DaVIE from a number of different formats, including R methylumi objects, CSV table formats, Genome Studio output files, and JSON based data. To pre-process our data, we first import raw data into Illumina's Genome Studio and use the built-in tool for background subtraction. Next we export the corrected data into R, and use the Lumi package for color correction to account for the two colors being used in the array data. This corrected data is then imported into DaVIE via command line scripts and further project-specific analyses can be performed in R. A web-interface import script is also available for data sets that have been color corrected in Genome Studio, and not further processed in R.

Since one of DaVIE's important advantages is the ability to compare data points across projects, important decisions about pre-processing must be made before data deposition. Using different methods for quality control and normalization can result in apparent disparities across projects that are out of proportion to the actual differences. All of these are currently controlled by established methods on an individual project level, but for large-scale data deposition, certain considerations are required. When comparing across projects in a large data set, adjustments, such as quantile normalization, used on an individual project risk over-correction that could introduce project specific artifacts. Such artifacts could subsequently appear as significant differences or similarities where none should otherwise appear. Consequently, it is important that the data stored in the database not be overly processed before being entered for comparison. For our own projects, we have a consistent set of pre-processing steps before deposition of data into DaVIE, as we described above. We believe that background subtraction and color correction, which correct for between-sample technical differences, do not induce artifacts and are comparable across projects, and so we perform both before depositing the data. Given that current literature on probe type normalization of data from the 450k array has not yet reached a consensus, we currently do not normalize our data for probe type differences before deposition, since differences in normalization method within the database could lead to more batch effects than actually exist. In the case where a particular effect can explicitly be identified and corrected, it is possible to perform modifications to the data before uploading it to the database, although such effects should be demonstrated to exist before imposing corrections. As with any database, however, individual users should be careful to ensure that data deposited is of good quality and uniformly processed across projects.

### 3.2. Data storage

Samples in DaVIE are collected into projects, a virtual grouping that describes the origin of the sample rather than any common trait of the samples themselves. For instance, a project may be a group of samples downloaded from GEO, the output of a single research lab or a series of samples collected for a single purpose. This structure is maintained throughout DaVIEs web interface and accompanying database, as sample data for a single project will frequently contain a consistent set of variable descriptors (e.g., age, sex, BMI). This modular format means that each project is maintained in its original integral form, allowing for analysis both within and between projects in DaVIE.

#### 3.2.1. Beta values

Beta values corresponding to the DNA methylation values of each probe on the Infinium HumanMethylation450 BeadChip (Illumina, San Diego, CA) are stored in the MongoDB database in a single collection. Because each sample provides values for 485,577 locations or probes for specific features in the genome, the number of data points in a project increases linearly with the number of samples collected with the array. However, grouping the samples by project reduces the number of documents (or rows) in the collection, allowing the collection to scale with the number of projects stored in the database, rather than the number of samples contained in the database. This, in turn, reduces the sizes of the indexes held by the database, making queries faster and more efficient. An example of a record in the methylation collection is provided in the Supplementary Material.

While each probe yields both *M*-values, a log scale measurement of methylation values ranging from −∞ to +∞, which are more frequently used for statistical analysis, and beta values, ranging from 0 to 1 representing unmethylated and methylated states, respectively, we have chosen to use beta values exclusively since they are more intuitive for the visualization process (Du et al., 2010).

#### 3.2.2. Sample variables

Outside of the DNA methylation data values themselves, the variables collected for each study are also necessary in order to place the DNA methylation data in context. Once integrated into the database, the variables for each sample are referred to as metadata. The storage of the metadata takes advantage of the JSON storage platform of the MongoDB database by storing key-value pairs for each sample. This allows samples to store unique information for samples that would otherwise be difficult to store in a table-based format, where the columns must be pre-defined and consistent across projects. MongoDB databases allow the user to define the key variables (e.g., age, sex, disease status, etc.) based on the available information for a sample. If the data is not present, it is not necessary to use placeholder values or null values, as the key itself can simply be left out of the sample information. An example of the format used to store sample information is given in the Supplementary Material.

Although this approach is extremely flexible, it also places the responsibility of data management on the researcher supplying the data. For instance, if a user provides the key sxe instead of sex, the data stored in that field will not be available for queries searching on the correctly spelled sex field. Thus, utilities were included in DaVIE to provide the ability to update incorrectly spelled keys and values, such that the database can also be used to store and maintain sample data.

This approach also has a further limitation that data stored in the database may not always share keys across projects, making comparisons across the full data set in DaVIE challenging. However, the approach taken allows samples to be grouped by values stored in up to three keys within a single project. For instance, within a single project, one could stratify samples by age, sex, and treatment/control groups simultaneously. In contrast, comparisons across groups are only provided for groups based on the project name or tissue type where available. Both project name and tissue type require a specific key name (project and tissuetype, respectively) to be included in the sample information, and samples that lack those fields will not be displayed when those visualization representations are selected.

#### 3.2.3. Integration of annotations

**Figure 1:**
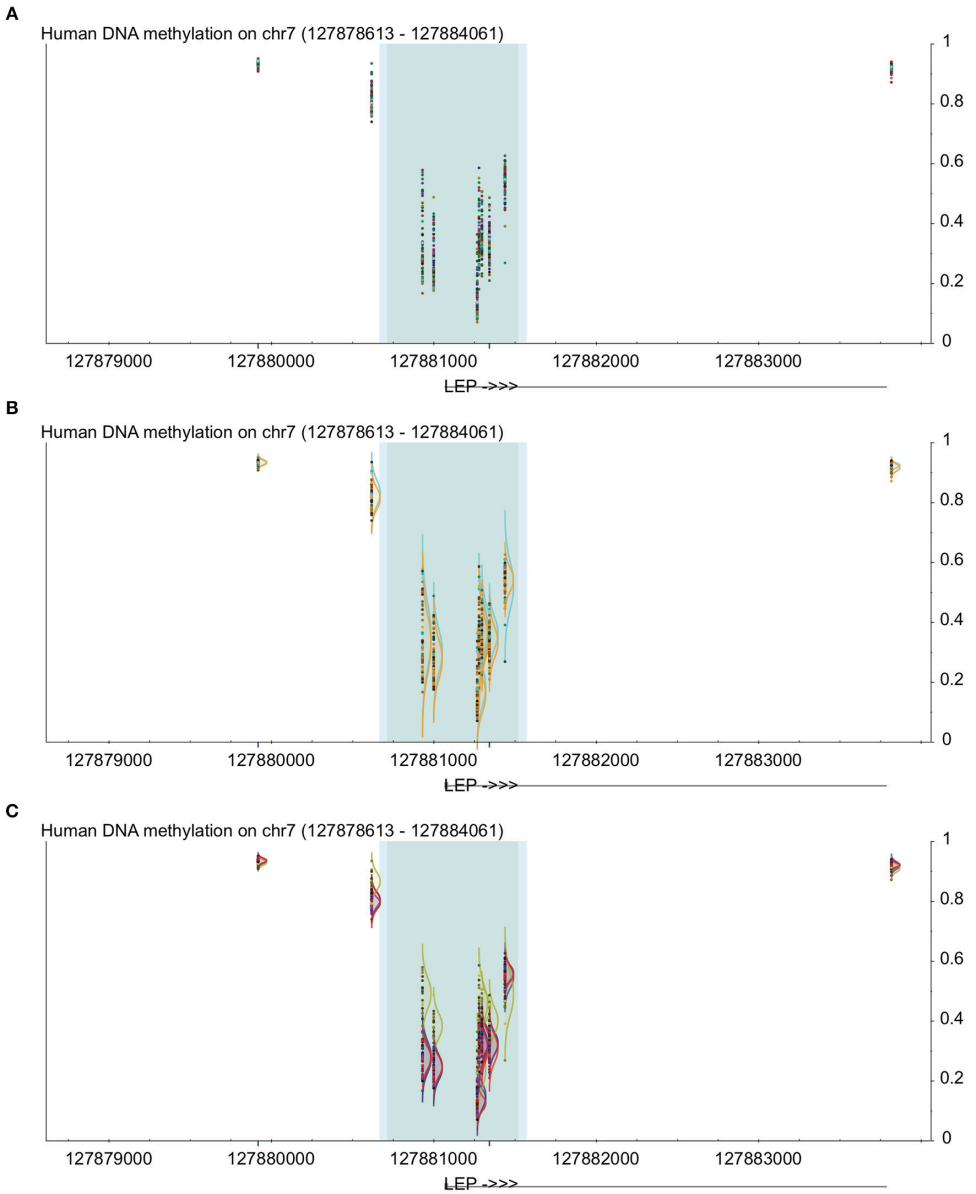
**Example of uses of visualization methods in DaVIE using LEP gene as an example**. All panels show DNA methylation from 0 to 1 on the y-axis at the LEP promoter, including high-density CpG islands (dark gray) and intermediate density islands (light blue), with gene structure indicated below. Dark blue lines below indicate unreliable probes. **(A)** Points view shows overall level and general distribution of methylation. **(B)** Grouped by sex, no obvious differences are observed. **(C)** Grouped by tissue, one of the four different tissues in this project shows clear distinction.

In order to interpret DNA methylation data, particularly across projects, it is very important to display it in the correct genomic context. To that end, we included two systems in DaVIE. The first was the inclusion of an independent re-annotation of probes on the 450k array (Price et al., 2013). This includes information on individual probes, such as the occurrence of SNPs within probes or at the CpG site of the probes themselves, which are displayed as blue and red squares just below the x-axis of the viewing area, respectively (Figure 1). This information can be critical when evaluating the distributions of DNA methylation observed for a specific probe, as the presence of a SNP within a CpG of a probe often results in patterns that reflect the distribution of the SNP instead of the methylation state of the CpG. Secondly, it is critical to be aware of the location of CpG islands and shores, in which DNA methylation is both reduced and in many cases more variable than non-island regions (Illingworth and Bird, 2009). Thus, these locations are taken from the same annotation source (Price et al., 2013), and displayed as a blue background behind the probe data points, using a deeper blue for the high-density islands, and a lighter blue for the intermediate-density islands.

#### 3.2.4. Data volume and extension

The use of a MongoDB database effectively allows an unbounded volume of storage, as the database may be “sharded” or fragmented over as many computers and storage systems as are available. Because the interface is provided for download, rather than being offered as a data hosting service, the amount of storage available at any given site will be determined by the resources provided for the database by the host institution.

Additionally, the database is also inherently flexible to other formats of data. Although the interface described here is applied exclusively to beta values for methylation arrays, extensions for storing other forms of epigenetic information can be implemented. A separate module is under development for chromatin Immunopreciptation data, and it would be possible, but not currently planned, to extend the database to include data from whole genome bisulfite sequencing or reduced representation bisulfite sequencing.

### 3.3. Interaction with the database

Users of the DaVIE interface are able to access data at a specific location in the database by using the controls provided through the navigation panel. Options provided include the organism (or database, if multiple data sets are kept in separate databases) of interest, the type of data (e.g., methylation), and what groups to impose on the data provided. A list of samples available for the organism (or database) are provided, along with an option to edit the metadata for a selected sample. Furthermore, controls are provided to directly access any region of interest in the genome, either by coordinate, or by probe id (e.g., cg08691422) or gene name (e.g., TP53). Options to change the visualization mode and hide samples are provided, as well, to allow finer control over the display.

### 3.4. Visualization

Unlike the track-based methods, DaVIE requires that information be deposited into to the attached database in order to display it. This was done explicitly to break away from the track-based methods commonly displayed by most bioinformatics databases. While track based information provides a lot of flexibility in allowing different types of information to be displayed along the same coordinate system, it becomes an obstacle for creating useful display when the data is all of the same type. By aligning the data in tracks, the ability to identify patterns within the data becomes significantly reduced. We have taken advantage of the fact that DaVIE is database-based to include a number of different visualization types.

#### 3.4.1. Visualization modes

Several visualization methods have been explored in DaVIE. With the ultimate goal of graphically representing relationships in the data, each visualization method attempts to illustrate a different aspect of the statistical relationship between the groups of samples.

The simplest visualization method is to show the raw data points, in place in the genomic coordinates. This visualization method can be suitable for identifying outlier samples or groups of samples that may influence other methods of analysis, but will also allow the user to identify patterns of DNA methylation across a genomic region, correctly lined up with the associated genomic features. By color coding the data points to the selected groups, the visual representation of the samples is simple to interpret—moreover, the visualization is interactive by providing information about each sample when the mouse is passed over a data point. This can allow identification of common patterns in the database, and identify groups that may deviate from the known pattern. Due to the density of data points displayed, this method is most effective when visualizing relatively small genomic regions.

Another built-in method of visualization is to show the distribution of each group (based on any criteria available within a single group or based on tissue type or project name across groups), which enables a direct comparison between any two or more groups of interest (Figures 1B,C). This can allow identification of common patterns in the database, and identify groups that may deviate from the known pattern. It can also be very useful when a CpG is found to be statistically significant within a project; by examining the site in the database, the user can quickly and visually determine whether the pattern observed in the specific project is common across many samples and tissues. For example, if a study shows that a specific CpG has lower levels of DNA methylation in participants with a disease vs. controls, then the user can examine all non-disease samples in the database to determine whether the controls in the study show typical distribution for the population as a whole. Since DaVIE is able to display samples by tissue type, this site can be examined in both the tissue of interest as well as other tissues, to determine whether the observed pattern in similar or different.

**Figure 2:**
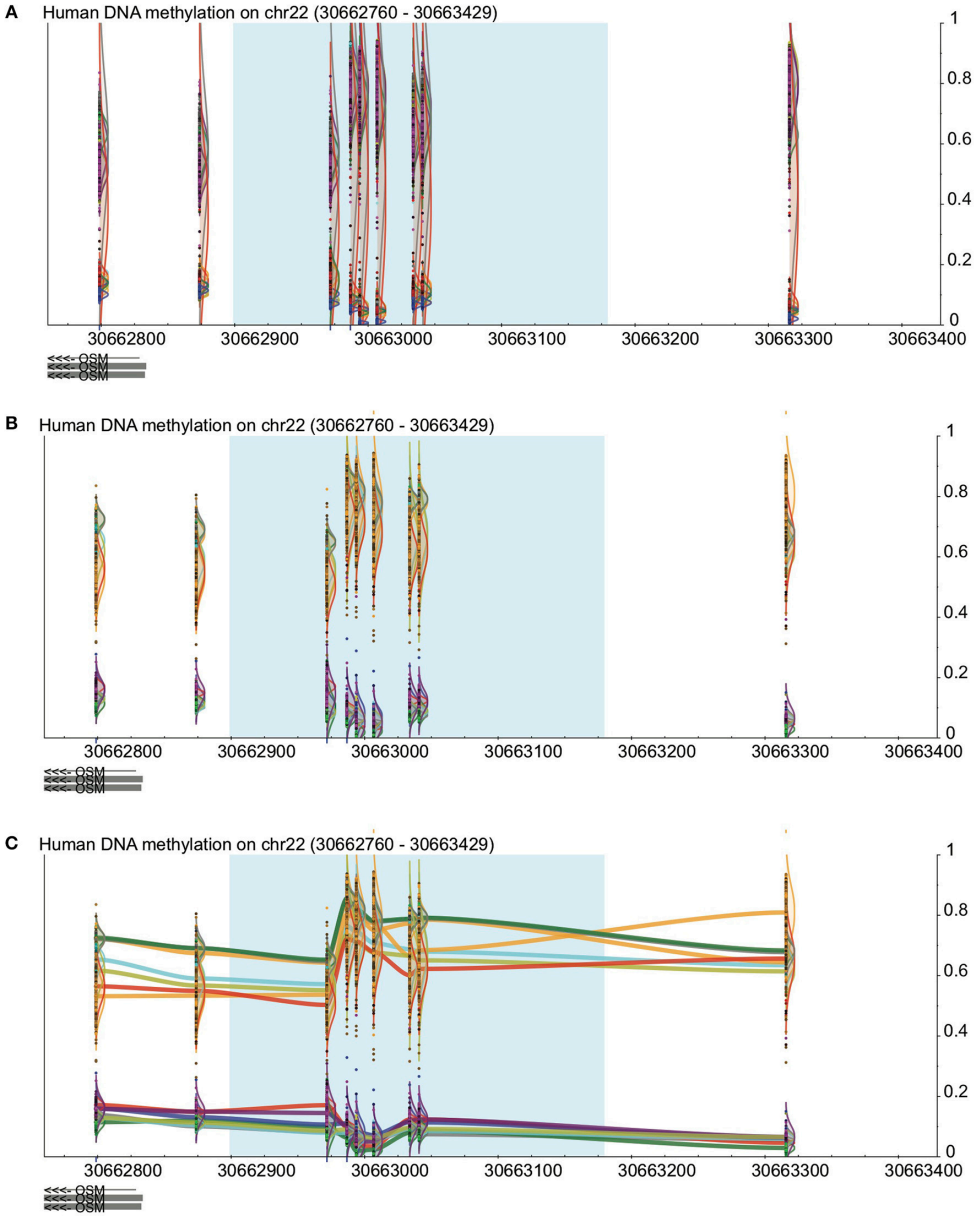
**Visualization in DaVIE across projects**. See Figure 1 for key. **(A)** In a total of over 500 samples in 12 different tissues, distinct groups are observed but separation is not achieved when the samples are colored by project. **(B)** When colored by tissue type, separation between tissues is improved. **(C)** With the trace view, subtler differences between the tissues are made visible.

In many cases, probe-by probe analysis only tells a part of the story, as DNA methylation trends often occur in broad regions around the genes that they play a part in regulating. Thus, the third visualization method built in to DaVIE shows DNA methylation as a smoothed line, connecting the probes for which information is available for a given group or project data set (Figure 2). In this setting, it is easy to identify trends across tissues. With each line representing a different tissue present in the projects selected, it is possible to quickly observe in which tissues high and low DNA methylation are present. Unlike applications such as bumphunter (Jaffe et al., 2012), no attempt is made to fit a linear model to the data set, and thus the visual impression may be sharp or jagged as the fluctuations in the trends necessitate. Importantly, the line joining CpGs should not be interpreted as imputed gradients of DNA methylation between CpGs, as the concordance of CpGs over genomic distances is highly variable and dependent on CpG density. The trace view should be used as a visualization tool only.

### 3.5. Validation using the X chromosome

**Figure 3:**
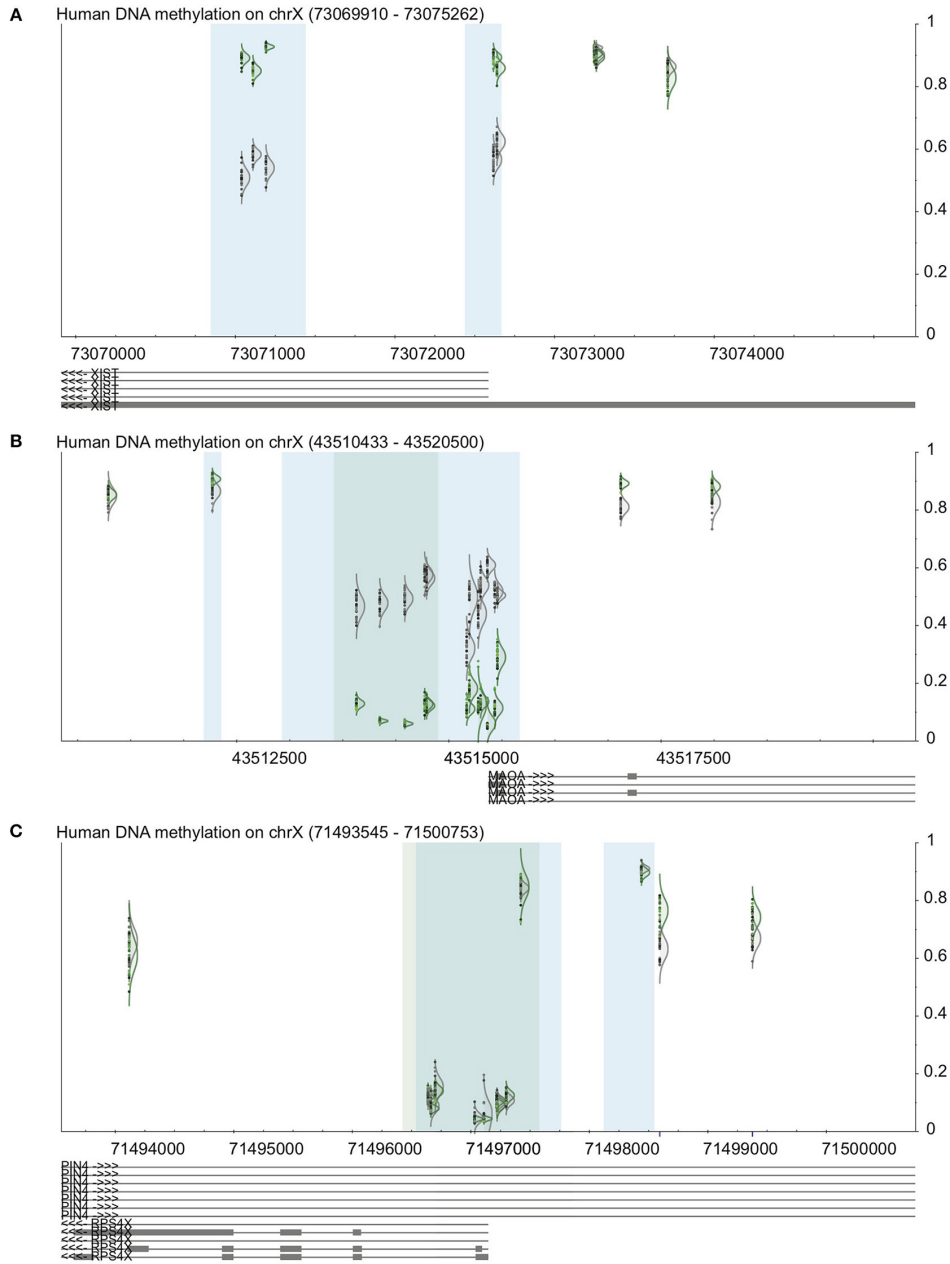
**Validation of visualization methods using examples from the X chromosome**. See Figure 1 for key. In all panels, female samples are in gray and male samples in green. **(A)** XIST promoter shows high methylation in males and approximately 50% in females. **(B)** MAOA, a gene subject to XCI, shows 50% methylation in females and low methylation in males. **(C)** RPS4X, a gene that escapes XCI, shows low island methylation regardless of sex.

We used DaVIE to examine promoters of genes on the X chromosome, which are known to have sex-specific and consistent patterns of DNA methylation (Yang et al., 2011). In a project with 46 blood samples (22 females and 24 males), we examined the promoter-associated CpG island and surrounding CpGs in the XIST gene, a gene that is subject to X inactivation, and a gene that escapes X inactivation. The patterns observed are consistent with known DNA methylation patterns of these genes (Figure 3). XIST shows approximately 50% methylation in females, and more than 75% in males at the two promoter associated CpG islands, but little difference between the sexes at non-island CpGs. MAOA, which is subject to X inactivation, also shows 50% DNA methylation in females, but very low methylation in males at the island, and also little difference at the non-island CpGs. The differences between male and female DNA methylation are more apparent at the high-density island (dark blue) than the intermediate-density island (light blue). Finally, RPS4X, which escapes X inactivation, shows typical low DNA methylation at the promoter-associated island and high methylation outside the island, which mostly does not different between males and females. Two probes just outside the CpG island do appear to be differentially methylated between males and females, but the blue line below each of the probes indicates that they contain a SNP, which could make them less reliable. This finding, then, should be confirmed by an independent method.

## 4. Discussion

DaVIE was developed in order to have a single platform through which DNA methylation data from a variety of tissues, projects, or platforms can be integrated and visualized. The goal was to be able to easily observe patterns in DNA methylation in a genomic context across projects, tissues, and diseases, which has allowed us to compare specific sites and patterns we observe in individual projects to a large number of other samples. With data deposited from both our own projects and from GEO, we have been able to use DaVIE to examine trends in DNA methylation at specific CpG sites or genes in over a 1000 samples. Since the data is displayed according to genomic location, all generations of the Illumina platforms including GoldenGate, 27k, and 450k can be displayed side-by-side. Because the data is relatively unprocessed, it is easy to compare across groups, and the structure of the database provides rapid access to both large collections as well as subsamples of the data stored. Thus, verification of hypotheses generated by other software packages, generation of new hypotheses based on DNA methylation patterns at specific genes or sites of interest, as well as unstructured exploratory investigations of a single data set alone or in the context of other large collections can be done interactively. The visualizations generated by the web front-end of the database are intuitive and informative, making trends and patterns obvious to even a casual observer.

### 4.1. DaVIE application stack

The web interface presented here represents a mix of technologies, some of which are generally common in bioinformatics applications, such as Django and Scalable Vector Graphics. In contrast, the back end, providing the data management, is housed in a MongoDB database, which is poorly represented in the bioinformatics literature. MongoDB databases provide an excellent structure for non-transactional data storage, which is ideal for databases in which few writes, but many reads take place. The non-transactional nature of the database results in an increase in database performance, as transactions are not necessarily blocking. MongoDB databases also provide an excellent platform for large volume storage, including the ability to distribute the data across many machines in a process called sharding. Although not employed here, sharding can provide a layer of scaleability that is difficult to achieve with most traditional SQL databases.

The use of scalable vector graphics also provides many advantages over the use of most image formats. By design, vector graphics are descriptions of the images they represent, rather than a fixed image. For instance, a circle is represented by the key word circle, with a set of properties describing the color and position of the circle. This, first, allows the graphics to be rendered by any client that is capable of interpreting the image description, and, also means that the image can be easily zoomed in and out without loss of quality. The former means that the server does not spend time rendering the image, but allows the web browser to do the work, generating the image observed by the user. The later mean that the image, once generated, can be used for high quality illustrations and can be manipulated easily by a user who would like to customize the image.

### 4.2. Data management

One of the major trends of the past decade in bioinformatics genome browsers has been the introduction of tracks, separate data sets that can be placed sequentially, aligned by a common coordinate set. This holds constant a single dimension, usually in the horizontal direction, representing the genome location, and the second, vertical, axis is allowed to be unique for each track because there is no overlap in the data presented. This method has allowed for innovations such as the ability to present user-supplied data sets alongside data maintained in a database, as well as for visualizing separate data sets in different ways, side-by-side. For many applications, this is clearly the direction in which bioinformatics visualization platforms will continue to progress.

For the DNA methylation database interface, where the visualization is limited to a single type of data, tracked browsing would make it more difficult to compare trends across data sets. By creating a single visualization area in which all data sets are presented, it is possible to directly compare values across projects in two dimensions simultaneously. This effectively superimposes each project, allowing even small differences in scale in either dimension to be instantly visible to the user.

Taking advantage of the ability to identify small variations requires that the data be easily grouped by traits of interest. In this case, any variables provided about each sample (e.g., phenotype, sex, age) can be used to group samples within a single project or across projects if the same variable is provided in more than one project.

For larger projects with complex data sets, it is often of great interest to ask questions about several descriptors simultaneously, investigating the interaction between two or more variables of interest. For instance, a cancer project may include data about the tumor type and course of treatment of patients. Rather than investigating these separately, the interface provides a means of generating compound groupings, in which up to three characteristics are provided. This allows the user to ask questions of specific sub groups, and to compare sub sets of the samples at a time (e.g., only lung cancers with any treatment type, or all cancers with a specific treatment type).

#### 4.2.1. Comparison of data across projects

The ability to compare data points across projects raises several important issues about the nature of the data being compared. Given that current literature on normalization of data from the 450k methylation array has not yet reached a consensus, it is important to be aware that small variations are possible between data sets that may not be significant. However, the visualization interface is designed around the comparison of single probes at a time, and makes it possible to identify outliers that can skew statistical comparisons.

Consequently, it is important that the data stored in the database not be overly massaged before entering the data for comparison. It is currently recommended not to use normalization processes that attempt to alter the distribution of probe values in aggregate. Probe-by-probe normalization, such as color correction, appears to be a reasonable process that does not introduce artifacts, but it is difficult to ensure that further normalization will not introduce batch effects greater than those they attempt to remove.

### 4.3. Additional utilities of DaVIE

The technology used to build DaVIE has, by design, a broad potential for expansion and application in other areas of research. Each of the database, web interface and visualization tools built could provide a foundation for further work, and the open-source availability of the source code encourages both future collaborative work as well as possibilities for extensions for other groups. The modular nature of the code also allows for each piece to be used individually to serve a specific purpose, or to be recombined with other software to build tools for new areas of research.

DaVIE provides an excellent reference model for groups looking for fast, schema-less methods of storing data for rapid retrieval. While not discussed here, it has been used for storing and retrieving ChIP-Seq and ChIP-chip data, and could easily be extended to process DNA sequencing data, genotyping data, or even expression levels as well. With the growth in next-generation sequencing, it may prove to be useful platform for further development.

DaVIE's web interface, being a simple Django web server, also serves as a template for future projects, as it could be used to rapidly prototype new visualization tools that bootstrap on top of the existing database and web server. For instance, prototype ChIP-chip and ChIP-Seq visualization has been undertaken as part of the web server. Other work to develop track-less visualization tools would easily be built on top of the existing infrastructure, replacing the visualization tools that are currently generated. This provides opportunities for work in any number of fields, such as visualizing the overlap of transcription factors, DNA-protein binding events or even comparing coverage between several sequencing samples. The existing web interface has provided interfaces for several methods for grouping and displaying the data, but could easily be expanded to include a wide array of tools, such as displaying discrete data distributions (e.g., age, height, weight) as gradients, making trends easier to identify.

Finally, the visualization engine used to generate SVG images is easily expandable, with the abiltity to quickly add and test new methods of visualizing data. SVG-based images are simple to modify by adding new objects, such as the trace view, illustrated in Figure 2. New ways of grouping and illustrating the data are limited mainly by the density of information it is possible to discern in a single image.

Visualization of DNA methylation data is also an area that will benefit from future technology developments. For instance, it would also be possible to replace the SVG image generation module with an HTML5 canvas elements could provide the ability to interact with the data and explore DNA methylation landscapes in ways that may not be possible in a two dimensional environment. Thus, the interface developed here provides an excellent platform for experimenting with new methods of displaying complex epigenetic data in an interactive way. There remain many avenues to explore in creating new and more intuitive ways to make the data more accessible for scientists who would otherwise be limited to statistical packages that process, but fail to provide clear and insightful methods of visualizing and interpreting DNA methylation data.

## Funding

Funding for this project was provided by the R Howard Webster Foundation and the NeuroDevNet NCE.

### Conflict of interest statement

The authors declare that the research was conducted in the absence of any commercial or financial relationships that could be construed as a potential conflict of interest.
