# Supplementary material for: DaVIE: Database for the Visualization and Integration of Epigenetic data
Source: Front Genet. 2014 Sep 18;5:325. doi: 10.3389/fgene.2014.00325 (PMC4166999; doi:10.3389/fgene.2014.00325)
Supplement: Supplementary file 1 [file DataSheet1.PDF]

Frontiers in Genetics

Research Article
4 July 2014

DaVIE: Database for the Visualization and Integration of Epigenetic data.

Anthony P. Fejes [1,*], Meaghan J. Jones [1], Michael S. Kobor [1,2,3]

[1] *Centre for Molecular Medicine and Therapeutics, 950 West 28th Avenue, Vancouver, BC V5Z 4H4, Canada*
[2] *Department of Medical Genetics, University of British Columbia, 2329 West Mall, Vancouver, BC V6T 1Z3, Canada*

Correspondence*:
Anthony P. Fejes
Kobor Laboratory, Centre for Molecular Medicine and Therapeutics, 950 West 28th Avenue, Vancouver, BC V5Z 4H4, Canada, apfejes@gmail.com

Michael S. Kobor, msk@cmmt.ubc.ca

1 SUPPLEMENTARY TABLES AND FIGURES

```
{
    "_id" : ObjectId("532c6473b0e1463c309aadfb"),
    "b" : {
        "sample1" : 0.21350858783895732,
        "sample2" : 0.23218597338424396,
        "sample3" : 0.18175209014903676,
        "sample4" : 0.16087443946188337,
        "sample5" : 0.19813344153962092,
        "sample6" : 0.070205775549023,
        "sample7" : 0.06262013899157179,
        "sample8" : 0.22254351975015904,
        "sample9" : 0.13848299681190224,
        "sample10" : 0.15519006783565856,
        "sample11" : 0.06269874602507951,
        "sample12" : 0.04578516193247072,
        "sample13" : 0.06116369894750777,
        "sample14" : 0.19812104225059693,
        "sample15" : 0.18980534284211142,
        "sample16" : 0.180594219770208,
        "sample17" : 0.17584476413516226,
        "sample18" : 0.17523336161959027
    },
    "pid" : "cg123456789",
    "project" : "Liver Cancer Samples"
}
```


Figure 1. Example of data preserved in the methylation collection of the mongoDB database. Grouping by project reduces the overall number of documents in the MongoDB collection, which subsequently reduces the overhead of the indexes used to access the data, which in turn increases the speed with which records .


```
{
    "_id" : ObjectId("51fa8565b0e1467b942344d5"),
    "age" : 30,
    "handedness" : "Left",
    "percentage_briefpraxis" : 100,
    "project" : "Epigenetics Project Example",
    "sample_group" : "DS",
    "sample_section" : "R02C01",
    "sample_well" : "C02",
    "sampleid" : "patient id #1",
    "samplelabel" : "patient id #1",
    "sentrix_barcode" : 684659013,
    "sex" : "M",
    "test_date" : "31-Mar-2011",
    "tissuetype" : "buccal",
}
```


Figure 2. An example of the meta data or sample data storage for a single sample. The number or records stored in the sample collection always equal the number of samples for which data is stored.
